# Supplementary material for: Negative mpMRI Rules Out Extra-Prostatic Extension in Prostate Cancer before Robot-Assisted Radical Prostatectomy
Source: Diagnostics (Basel). 2022 Apr 23;12(5):1057. doi: 10.3390/diagnostics12051057 (PMC9139507; doi:10.3390/diagnostics12051057)
Supplement: Supplementary file 1 [file diagnostics-12-01057-s001.zip › diagnostics-1562897-supplementary.pdf]

Negative mpMRI rules out extra-prostatic extension before robot assisted radical prostatectomy.

Supplementary Online Material

File S1. Exclusion and Inclusion Criteria for the NeuroSAFE PROOF Trial (NCT 03317990).

Inclusion criteria:

1. Men opting to undergo RALP for organ confined prostate cancer.
2. Potent men (IIEF 22-25 not using PDE5i or other medications or devices).
3. Men who are continent of urine (no self-reported urinary incontinence).
4. Has given written informed consent.
5. Ability to read English sufficiently to answer questionnaires and understand PIS.

Exclusion criteria:

1. Unable to undergo robotic prostatectomy.
2. Known overactive bladder.
3. Previous treatment for prostate cancer.
4. Previous/current hormone treatment for prostate cancer.
5. Nerve sparing deemed futile due to locally advanced disease by surgeon and radiologist.

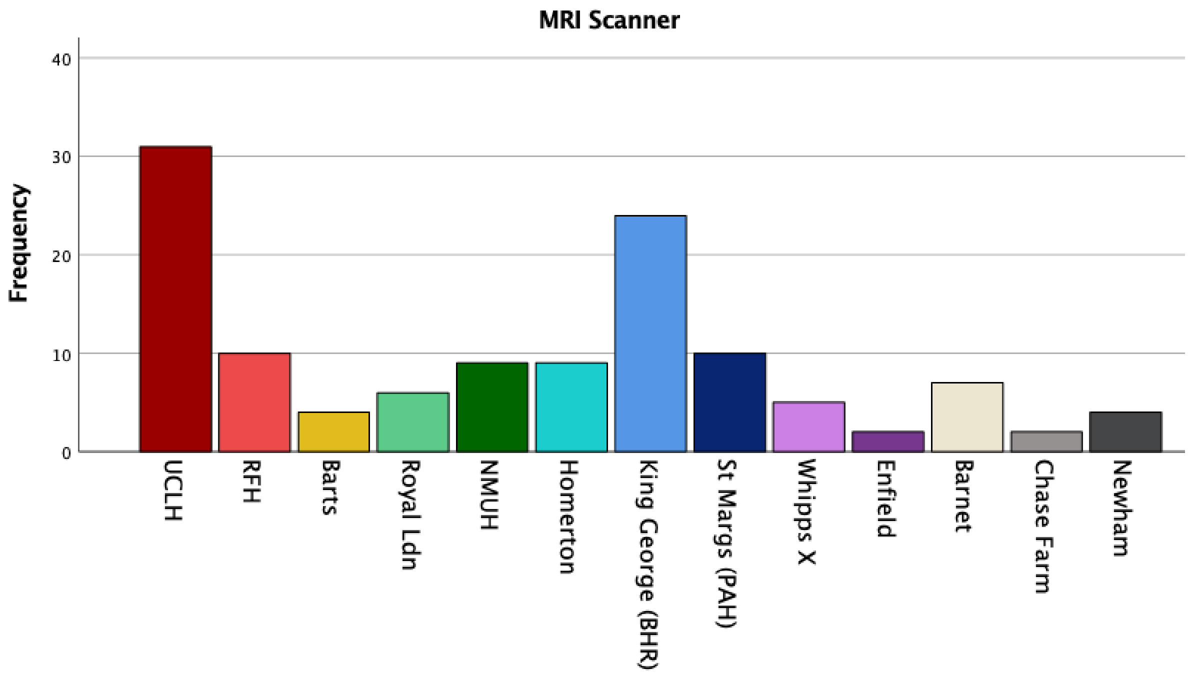

<u>Figure S1.</u> Locations of MRI Scanners and corresponding numbers of mpMRI Scans Performed. *Legend and numbers of scans performed in brackets; UCLH, University College London Hospitals (31); RFH, Royal Free London NHS Foundation Trust (10); Barts, St Bartholomew's Hospitals part of Barts Health NHS Trust; Royal Ldn, Royal London Hospitals part of Barts Health NHS Foundation Trust; NMUH, North Middlesex University Hospital; Homerton, Homerton University Hospital NHS Foundation Trust, King George (BHR), King George Hospital part of Barking, Havering and Redbridge University Hospitals NHS Trust; St Margs (PAH), St Margaret's Hospital part of The Princess Alexandra Hospital NHS Trust; Whipps X, Whipps Cross Hospital part of Barts Health NHS Trust; Enfield, Enfield Alliance MRI Centre performed on behalf of Chase Farm Hospital part of Royal Free London NHS Foundation Trust; Barnet, Barnet Hospital part of Royal Free London NHS Foundation Trust; Chase Farm, Chase Farm Hospital part of Royal Free London NHS Foundation Trust; Newham, Newham University Hospital part of Barts Health NHS Trust.*

CRF MRI Extra-Prostatic Extension (EPE) – Radical Prostatectomy Study
v1.4 18 December 2019

Patient Initials: *Date:*
Radiologist Initials: *Date of Scan:*
Hospital number:

Perceived likelihood of EPE: *(1, highly un- likely; 2, unlikely; 3, equivocal or indeterminate; 4, likely; and 5, highly likely.)*

(please report strictly in order and put a number (1-5) on each side.)

Right Left

T2

ESUR EPE criteria	R	L
Abutment (>10mm)		
Irregularity		
Neurovascular Bundle thickening		
Bulge, loss of capsule		
Measurable extra-capsular extension		

T2 + DWI

ESUR EPE criteria	R	L
Abutment (>10mm)		
Irregularity		
Neurovascular Bundle thickening		
Bulge, loss of capsule		
Measurable extra-capsular extension		

T2 + DWI + DCE

ESUR EPE criteria	R	L
Abutment (>10mm)		
Irregularity		
Neurovascular Bundle thickening		
Bulge, loss of capsule		
Measurable extra-capsular extension		

All sequences + PSA

All sequences + PSA + Biopsy information

Quality of Scan (1-5)	

NS Recommendation (Y/N)			
**Right**<br>Intra<br>Inter<br>Non		**Left**<br>Intra<br>Inter<br>Non	

Figure S2. Study Case Reporting Form.

<u>Table S1.</u> Summary for Assessment of the diagnostic quality of mpMRI scans using the PI-QUAL score.

<table>
<tr><th>PI-QUAL SCORE</th><th>Criteria</th><th>Clinical Implications</th></tr>
<tr><td>1</td><td>All mpMRI sequences are below the minimum standard for diagnostic quality</td><td rowspan="2">It is NOT possible to rule in all significant lesions.

It is NOT possible to rule out all significant lesions.</td></tr>
<tr><td>2</td><td>Only one mpMRI sequence is of acceptable diagnostic quality.</td></tr>
<tr><td>3</td><td>At least two mpMRI sequences taken together are of acceptable diagnostic quality.</td><td>It is possible to rule in all significant lesions.

It is NOT possible to rule out all significant lesions.</td></tr>
<tr><td>4</td><td>Two or more mpMRI sequences are independently of optimal diagnostic quality.</td><td rowspan="2">It is possible to rule in all significant lesions.

It is possible to rule out all significant lesions.</td></tr>
<tr><td>5</td><td>All mpMRI sequences are of optimal diagnostic quality.</td></tr>
</table>

T2-WI

Technical parameters	
Axial plane	
Sagittal or coronal plane	
Adequate field of view	
Adequate in-plane resolution	
Adequate slice thickness	
Z-axis correctly positioned	
**Visual assessment**	
Capsule clearly delineated	
Seminal vesicles clearly delineated	
Ejaculatory ducts clearly delineated	
Neurovascular bundles clearly delineated	
Sphincter muscle clearly delineated	
Absence of artefacts (e.g. movement)	

Is T2-WI of diagnostic quality?

☐ Yes
☐ No

DWI

Technical parameters	
Axial plane matching T2-WI	
Adequate field of view	
Adequate in-plane resolution	
Adequate slice thickness	
Multiple [> 2] *b* values acquired	
High *b* value (synthesised or acquired)	
**Visual assessment**	
Adequate ADC map	
Absence of artefacts (e.g. rectal air)	

Is DWI of diagnostic quality?

☐ Yes
☐ No

DCE

Technical parameters	
Axial plane matching T2-WI	
Adequate field of view	
Adequate in-plane resolution	
Adequate slice thickness	
Pre-contrast T1-WI available	
Fat suppression/subtraction	
Adequate temporal resolution [≤ 10 sec]	
Adequate total observation rate [≥ 2min]	
**Visual assessment**	
Capsular vessels clearly delineated	
Vessels in the Alcock's canal clearly delineated	
Absence of artefacts (e.g. movement)	

Is DCE of diagnostic quality?

☐ Yes
☐ No

PI-QUAL score:

1 ☐
2 ☐
3 ☐
4 ☐
5 ☐

Figure S3. Scoring sheet for assessing the quality of mpRMI using the PI-QUAL scoring system. T2-WI=T2-weighted imaging; DWI=diffusion-weighted imaging; DCE=dynamic contrast-enhanced; ADC=apparent diffusion coefficient. **Reproduced with permission of Giganti, et al. Prostate Imaging Quality (PI-QUAL): A New Quality Control Scoring System for Multiparametric Magnetic Resonsnce Imaging of the Prostate from the PRECISION trial. Eur Urol Onc; 3(202): 615-619.*

Table S2. Clinical and Radiological features of the 9 men who had mpMRI repeated following prostate biopsy.

Age, years (mean (IQR))	57.6 (50 - 59)
Reason repeat scan	
‘Previous scan poor quality’	2
‘For RP planning’	3
‘Repeat staging/old scan’	3
‘suitable for focal therapy?’	1
Time since previous scan, days (mean (IQR))	233 (76 - 353)
Time since biopsy, days (mean (IQR))	164 (63 - 475)
Time until RARP, days (mean (IQR))	13 (4 - 24)
Pathological stage	
pT2a	1
pT2c	4
pT3a	3
pT3b	1
PI-QUAL score	
3	3
4	6
Sensitivity, % (95% CI)	100 (73.5 - 100)
Specificity, % (95% CI)	63.2 (46 - 78.2)
AUC (95% CI)	0.93 (0.85 - 0.1)

*All repeat scans were performed at the academic hospital where RARP and MRI reporting was performed.

Table S3. Pathological EPE according to final Likert score by individual radiologist per prostate lobe.

	Reader 1 (%)	*Reader 2*[†] *(%)*	*Reader 3*[‡] *(%)*
Likert 1	0/0 (0)	0/8 (0)	0/13 (0)
Likert 2	5/121 (4.1)	3/68 (4.4)	4/92 (4.3)
Likert 3	7/50 (14)	11/90 (12.2)	11/68 (16.2)
Likert 4	16/38 (42.1)	19/50 (38)	18/40 (45)
Likert 5	15/22 (68.2)	7/9 (77.8)	9/11 (81.8)
Total	43/231	40/225	42/224

[†]Reader 2, 6 scores missing. [‡]Reader 3, 7 scores missing.

Table S4. Per patient analysis of Sensitivity (SE), specificity (SP), positive predictive value (PPV), negative predictive value (NPV) and area under the curve (AUC) by individual radiologist and combined where Likert score 3≥ was positive scan for pathological.

	Reader 1	*Reader 2*	*Reader 3*	*Readers Combined*
*SE**	97.5 (88.6-99.9)	100 (90.8-100)	94.9 (82.7-99.4)	97.4 (92.7-99.5)
*SP**	40.5 (21.9-51.8)	8.5 (3.5-16.8)	23.5 (13.8-38.2)	24.4 (19.2-30.3)
*PPV**	44.3 (33.7-55.3)	33.6 (25-43.1)	37.4 (27.9-47.7)	38 (32.5-43.8)
*NPV**	97.1 (85.1-99.3)	100 (59-100)	90.5 (69.6-98.8)	95.2 (86.7-99)
*AUC**	0.69 (0.6-0.78)	0.54 (0.44-0.65)	0.59 (0.49-0.7)	0.61 (0.55-0.67)

*95% confidence intervals in parentheses.

Table S5. Inter-reader agreement (κw) for Likert based mpMRI assessment of EPE.

Readers	T2WI only	+DWI	+DCE	+PSA	Final Read†
**1 & 2**	0.66<br>(0.57 - 0.74)	0.63<br>(0.54 - 0.72)	0.6<br>(0.51 - 0.7)	0.64<br>(0.56 - 0.72)	0.62<br>(0.54 - 0.7)
**1 & 3**	0. 64<br>(0.48 - 0.65)	0.57<br>(0.48 - 0.67)	0.58<br>(0.48 - 0.67)	0.6<br>(0.51 - 0.69)	0.64<br>(0.56 - 0.72)
**2 & 3**	0.56<br>(0.48 - 0.65)	0.54<br>(0.45 - 0.63)	0.55<br>(0.45 - 0.65)	0.59<br>(0.51 - 0.67)	0.64<br>(0.57 - 0.71)

†Including information from the prostate biopsy.

Data are weighted Kappa coefficient (κw) statistics and data in parentheses are 95% confidence intervals. Traditional conventions; Kappa (K) <0 indicates no agreement; K= 0-0.2, slight agreement; K = 0.21-0.4, fair agreement; K = 0.41-6, moderate agreement; K = 0.61-0.8, substantial agreement; K = 0.81-1, almost perfect agreement. All analyses were performed using SPSS software (version 27, IBM).

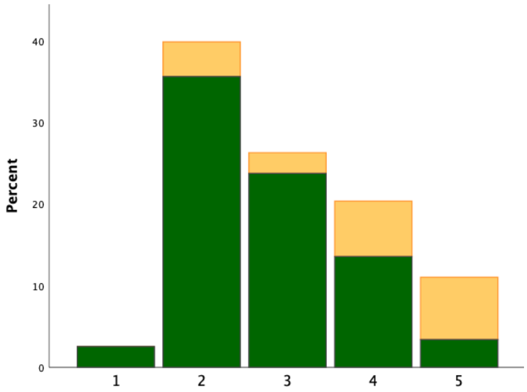

A. Biparametric MRI scans.

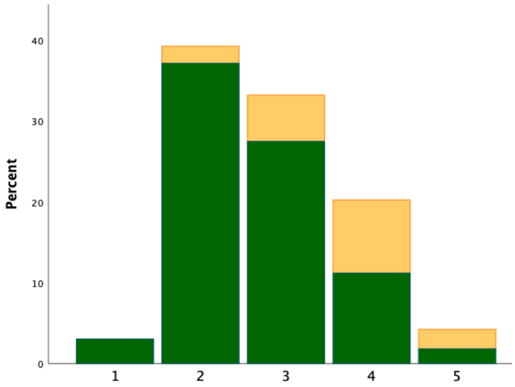

B. PI-QUAL score 1-3 mpMRI scans.

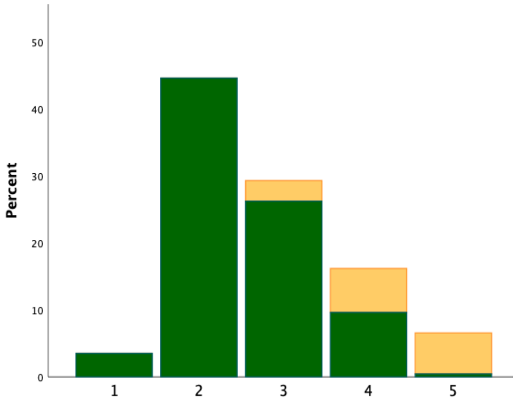

C. PI-QUAL score 4-5 mpMRI scans.

<u>**Figure S4.**</u> Stacked bar charts showing proportion clinical detection of EPE per lobe according to Final Likert score for biparametric scans (A), PI-QUAL score 1-3 scans (B), and PI-QUAL score 4-5 scans (C), respectively.

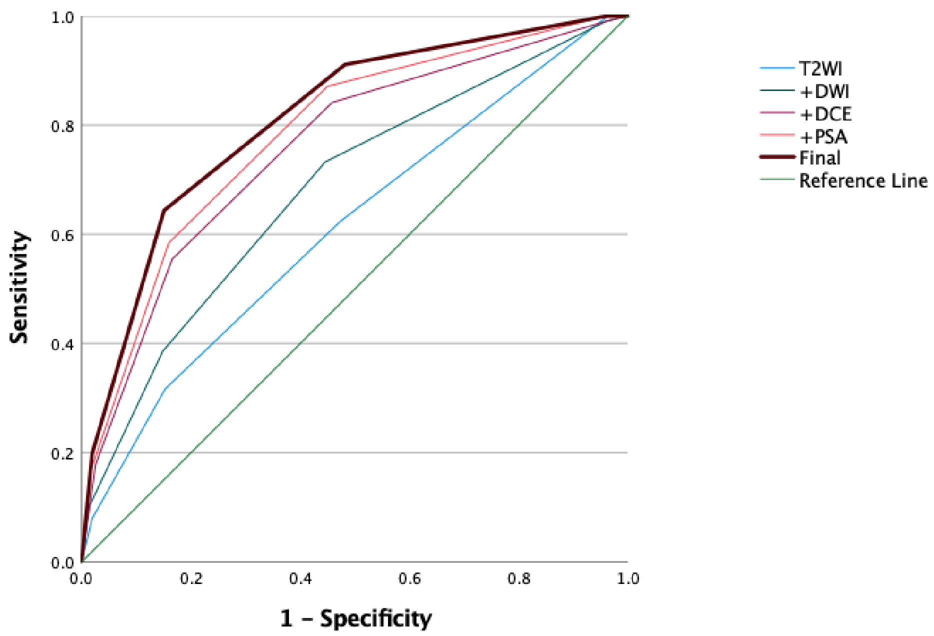

<u>**Figure S5.**</u> ROC curves for prediction of pathologic EPE using mpMRI for all 3 readers with additional imaging and information in sequence. **Area under the Curve (AUC)** (with 95% CI), T2WI 0.62 (0.56, 0.69); +DWI 0.69 (0.64, 0.75); +DCE 0.76 (0.71, 0.82); +PSA 0.79 (0.74, 0.84); Final 0.82 (0.77, 0.86). Legend: T2WI, T2 weighted-imaging; DWI, diffusion-weighted imaging; DCE, dynamic contrast enhanced; PSA, prostate specific antigen; Final (including biopsy information).

Table S6. Visual features according to radiological interpretation of positive scan for EPE (Likert ≥3).

	Abutment	Irregularity	NVB Thickening	Bulge	Measurable ECE
**Likert ≥3**	57.9%	36%	5%	20.1%	6.6%
**Likert 1-2**	17.2%	5.3%	0%	4.6%	0.3%
Overall	*39.9%*	*21.9%*	*2.7%*	*13.1%*	*3.7%*

Abbreviations: EPE=extra-prostatic extension, NVB=neurovascular bundle, ECE=extra-capsular extension.

Table S7. Visual features according to EPE on final pathological assessment of RP specimen.

	Abutment	Irregularity	NVB Thickening	Bulge	Measurable ECE
**EPE present**	64.3%	40.5%	6.3%	30.2%	11.1%
**No EPE**	34.6%	18.1%	1.9%	9.3%	2.1%
Overall	*39.9%*	*21.9%*	*2.7%*	*13.1%*	*3.7%*

Abbreviations: EPE=extra-prostatic extension, RP=radical prostatectomy, NVB=neurovascular bundle, ECE=extra-capsular extension.

<u>Table S8.</u> Diagnostic Performance of mpMRI when Likert score ≥4 for final read indicates positive diagnostic test.

	**Reader 1 (%)**	**Reader 2 (%)**	**Reader 3 (%)**	**Readers combined (%)**
**SE**	72.1 (56.3 - 84.6)	65 (48.3 - 79.4)	64.3 (48 - 78.5)	67.2 (58.2-75.3)
**SP**	84.6 (78.6 - 89.4)	82.2 (75.9 - 87.4)	86.8 (81 - 91.4)	84.5 (81.2-87.4)
**PPV**	51.7 (38.4 - 64.8)	44.1 (31.2 - 57.6)	52.9 (38.5 - 67.1)	49.4 (41.7 - 57.2)
**NPV**	93 (88.1 - 96.3)	91.6 (86.3 - 95.3)	91.3 (86.1 - 95.1)	92 (89.3 - 94.2)

95% confidence intervals in brackets.
